# Case Report: How an Iliac Vein Lesion During Totally Endoscopic Preperitoneal Repair of an Inguinal Hernia Can Be Safely Managed

**DOI:** 10.3389/fsurg.2021.636635

**Published:** 2021-08-12

**Authors:** Navid Tabriz, Verena Nicole Uslar, Timur Cetin, Andreas Marth, Dirk Weyhe

**Affiliations:** ^1^University Hospital for Visceral Surgery, Pius Hospital Oldenburg, University of Oldenburg, Oldenburg, Germany; ^2^Department for Anesthesiology, Pius Hospital Oldenburg, Oldenburg, Germany

**Keywords:** groin hernia, TEP, TAPP, PEEP, CO_2_ pressure, iliac vein injury

## Abstract

Inguinal hernia repair is a common surgical procedure with an acceptably low complication rate. However, complications with potentially life-threating consequences may occur in rare cases. These complications might be very challenging to manage, even more in laparo-endoscopic interventions compared to open repair. One of these challenges can be the treatment of an intraoperative injury to the iliac vein. To the best of our knowledge, a lesion of the iliac vein during TEP (totally endoscopic preperitoneal) for inguinal hernia repair, and a safe technique for its management have not been reported yet. We report the case of a 75-year-old male patient with previous abdominal surgery scheduled for TEP repair of an inguinal hernia. During surgery, the iliac vein was damaged. If we had performed a laparotomy in this situation, the potentially life-threatening condition of the patient could have deteriorated further. Instead, to avoid a potential CO_2_ associated embolism, the preperitoneal pressure was gradually reduced, and the positive end expiratory pressure (PEEP) was increased in the manner that a balance between excessive bleeding and potential development of a CO_2_ embolism was achieved. The injured vein was sutured endoscopically, and in addition a hemostatic patch was applied. We then continued with the planned surgical procedure. Thrombosis of the sutured vein was prevented by prophylactic administration of low molecular weight heparin until the 14th postoperative day. We conclude that in case of major vein injury during TEP, which might happen irrespective of prior abdominal surgery, the preperitoneal pressure and PEEP adjustment can be used to handle the complication.

## Introduction

Inguinal hernia surgery is one of the most common surgeries worldwide and it is performed on more than 20 million people annually (1). In adults, mesh repair techniques are recommended as first choice, either by an open or a laparo-endoscopic repair. According to the Hernia Surge and the IEHS guideline, an open anterior approach is favorable in patients with prior abdominal surgery (2, 3). However, also in accordance with the same guidelines, the choice of surgical procedure can be based on surgeon's expertise and a laparo-endoscopy can be chosen in patients after previous transabdominal radical prostatectomy (2, 3).

Due to faster recovery time, lower chronic pain sensations, and cost-effectiveness, laparo-endoscopic reparation-procedures such as TAPP (transabdominal preperitoneal hernioplasty) or TEP (totally endoscopic preperitoneal hernioplasty) are often preferred over open techniques. However, these surgeries can cause complications such as visceral injury or hemorrhage, especially out of the so-called corona mortis, that are unlikely to happen in open repair techniques (4, 5). Severe bleeding in laparo-endoscopic groin hernia surgery is rare and is reported with an incidence of 0.1–0.4% (6, 7).

However, if an intraoperative venous injury is detected, the management can be very challenging. We describe a technique for the treatment of an iatrogenic laceration of the iliac vein in a TEP repair of an indirect groin hernia, and discuss the proposed technique in the light of benefits and drawbacks of TEP and TAPP procedures.

## Case Presentation

A 75-year-old male patient presented with a suspected symptomatic groin hernia on the right side. Standard diagnostic routine was performed and the patient was scheduled for elective surgery in TEP technique. During consultation, the patient reported an incidence of prostatic cancer, which was removed by median laparotomy in 2003, and an appendectomy, which was done via McBurney's incision in the childhood. The patient was classified as ASA III with the following pre-existing conditions: bronchial asthma, circulatory disorder of the eye, and various allergies. Otherwise, according to preoperative diagnostics the patient was in good health. In accordance with the relevant guidelines, we opted for TEP instead of a Lichtenstein procedure, since this is the preferred technique in our clinic, and is performed more than 800 times/year.

**Figure 1:**
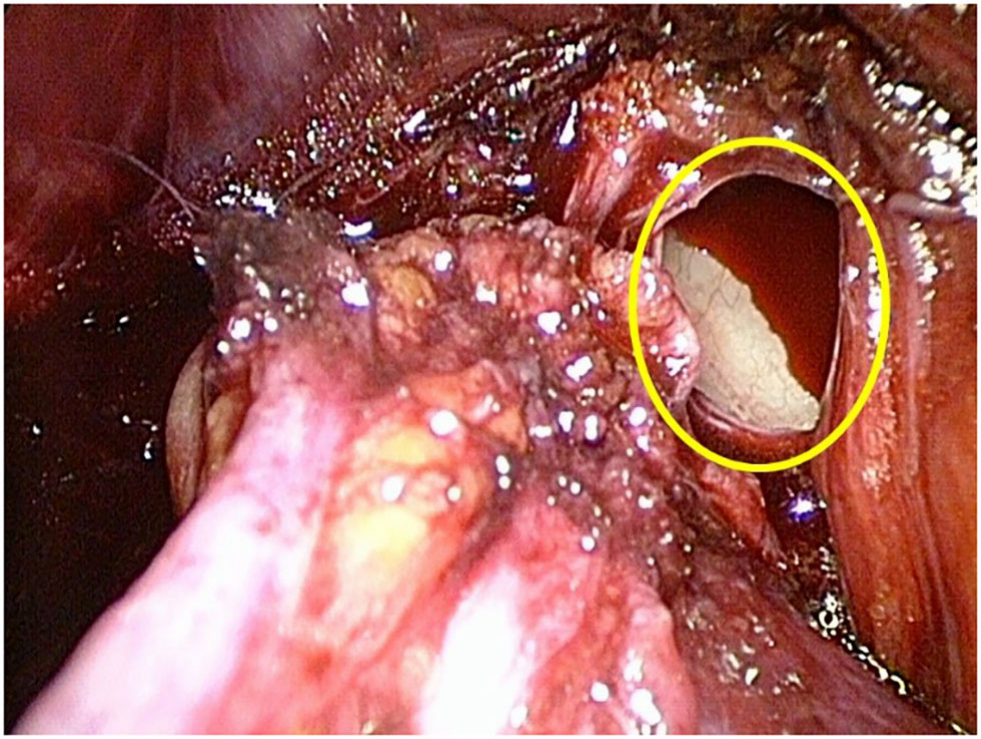
Intraoperative view of the open right iliac vein (yellow oval), despite the injury no bleeding is seen due to the preperitoneal pressure of 12 mmHg.

**Figure 2:**
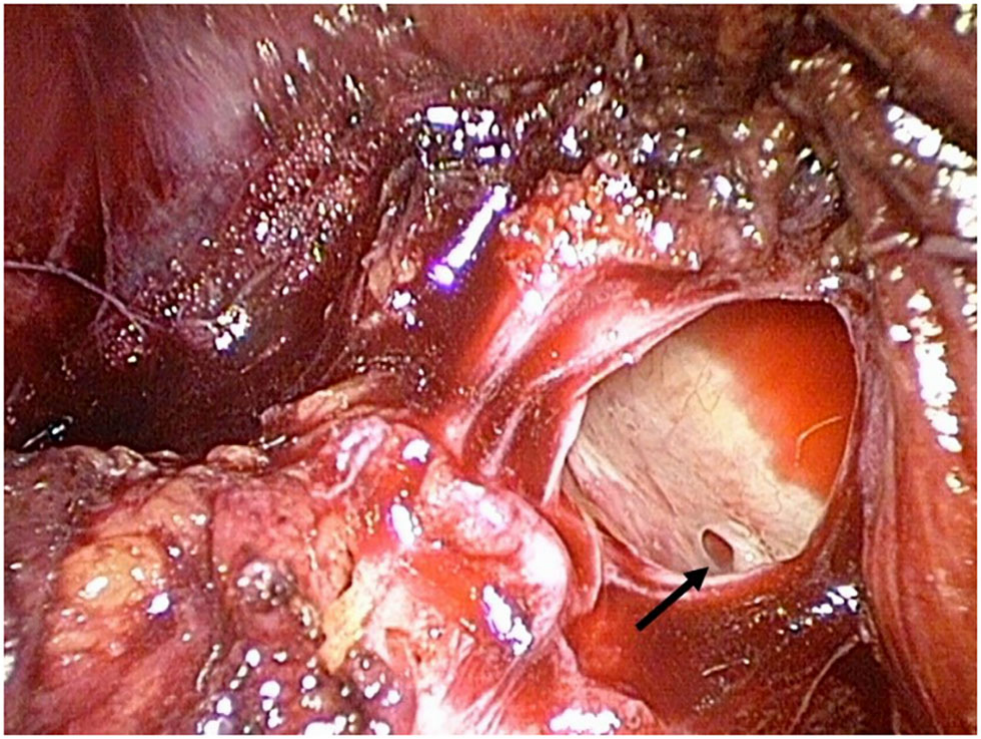
The preperitoneal pressure is increased to 14 mmHg; the intraluminal blood volume is now more compressed and a venous branch is visible through the injured lumen (black arrow).

TEP repair was performed with general anesthesia through three ports, an optic infraumbilical port and two surgeon's ports, one in the midline halfway between the umbilicus and symphysis, and a second one on the level of the umbilicus in the line of right anterior superior iliac spine. The preperitoneal space was inflated with a pressure of 12 mmHg with warmed carbon dioxide. After extensive adhesiolysis of scar formation due to the previous surgery in the midline, the Retzius space was prepared. The hernia sac was dissected from the spermatic cord and its vessels, and enough space for mesh placement was gained laterally. Before mesh placement, the preperitoneal space was controlled to identify potential bleeding manifestations. A one-centimeter sized laceration of the iliac vein was detected. Due to the preperitoneal CO_2_ pressure of 12 mmHg an active bleeding of the injured vein could not be seen (Figures 1, 2). Therefore, the pressure was reduced to 8 mmHg to ensure that the injured structure was actually the vein. Using this maneuver, an active bleeding out of the vein was verified and the pressure was raised immediately to 12 mmHg to avoid blood loss.

**Figure 3:**
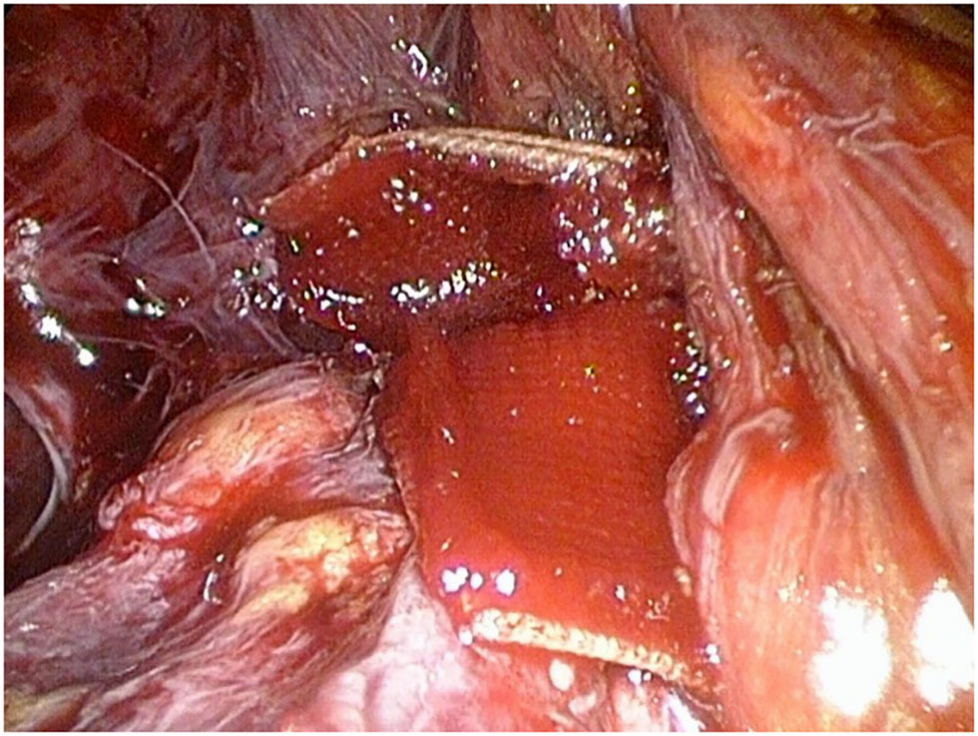
The defect is sutured (not shown) and additionally covered by a hemostatic patch (Veriset, Covidien).

**Figure 4:**
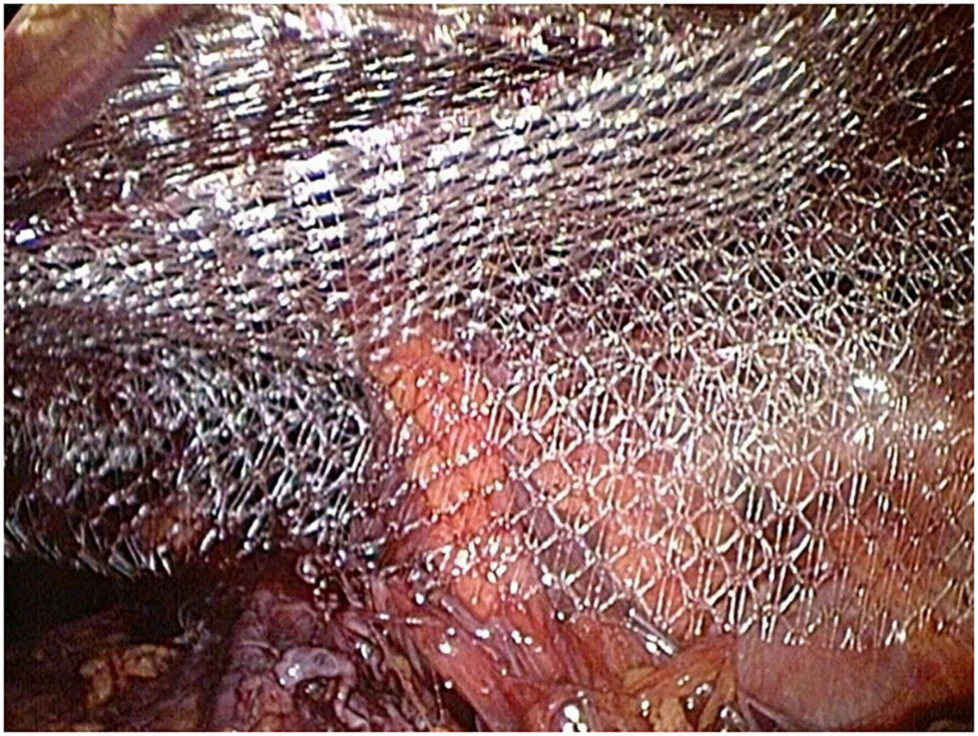
The inguinal hernia is supplied with polypropylene mesh in double mesh technique (Bard, SoftMesh).

At this stage, the entire operation theater staff was briefed on the potential life-threatening situation. Close communication with the anesthesiologist was indispensable for further action. To avoid a potential CO_2_ associated embolism, the preperitoneal pressure was gradually reduced to 8 mmHg and the positive end expiratory pressure (PEEP) was increased and then constantly adapted to values between 5 and 12 mmHg in such a manner that excessive bleeding as well as the development of CO_2_ embolism could be avoided. The injured vein was sutured endoscopically. In addition, a hemostatic patch (Veriset, Covidien) was applied (Figure 3). Then the preperitoneal pressure was reduced to 8 mmHg to check the stability of the suture, followed by mesh placement of a 15 x 12 cm large pore monofilament polypropylene mesh (Bard Soft Mesh), according to in-house standards and previously described by Weyhe D. et. al in double mesh technique without fixation (8) (Figure 4). A prophylactic 12 mm easy-flow drain was introduced into the retropubic area. The intraoperative blood loss was approximately 150 ml, and the surgery time was 70 min. In the postoperative course, there were no further complications. The drain was removed on the second postoperative day, and the patient was discharged at the 4th postoperative day. To prevent a thrombosis of the sutured vein, a prophylaxis with low molecular weight heparin until the 14th postoperative day was carried out. Neither surgery-related complications nor thrombosis of the iliac vein could be detected after a follow-up of 4 weeks.

During the last follow-up performed almost 4 years after the surgery, the patient reported no further problems, neither on the operated right side, nor on the left side. The Carolina Comfort Scale (9) showed no pain or foreign body feeling whatsoever during any of the enquired activities.

A time line of relevant events is shown in Table 1.

**Table 1:** Timeline of relevant diagnostics and therapies.

**Date**	**Diagnostics or therapy performed**
Childhood	Conventional appendectomy
2003	Prostatectomy
unknown	Varicose vein surgery
07.06.2016	Presentation in an outpatient clinic with well repositionable inguinal hernia on the right side
15.06.2016	Physical examination: vesicular breathing sounds on both sides, heart sounds inconspicuous, abdomen soft
15.06.2016	ECG: normal
16.06.2016	Elective TEP of the right side in double mesh technique with Bard-Soft-Mesh (9 x 14 cm & 6 x 14 cm); Opening of the right external iliac vein, endoscopic suture with V-LOC 3.0, application of a hemostatic patch (Veriset, Covidien) and a 12 mm Easy Flow drain, sandbag. Cut-suture time: 70 min.
16.06.−17.06.2016	Intermediate Care Station: Drainage delivers 100 ml of bloody-serous fluid
17.06.2016	Color-coded duplex sonography of the pelvic-leg veins on the right: V. femoralis communis properly flowed through
20.06.2016	Discharge in a complaint-free state. low molecular weight heparin (Clexane, Sanofi) 40 mg continue until 07.07.2016
05.07.2016	Control appointment. Patient reports no complaints
02.11.2018	Follow-up in our outpatient clinic with a discreetly symptomatic inguinal hernia on the left side. Decision for watchful waiting.
02.03.2020	Telephone contact. No current complaints. Patient receives consent form and Carolina Comfort Scale questionnaire by mail.
19.03.2020	Second telephone contact initiated by the patient to clear up some questions
25.03.2020	Questionnaire and consent form received back

## Discussion and Conclusion

This case report was prepared with the intention to present a way of managing a potentially life-threatening complication, which can occur anytime in TEP technique, even in primary hernia repair without prior abdominal surgery. In our institution, the TEP technique will be the primary method in all patients, if general anesthesia is tolerable. Even in cases of recurrent hernia after TEP and TAPP or previous pelvic operations, we initially will explore the preperitoneal space to decide if a TEP procedure is feasible, or if a conversion to open hernia surgery (Lichtenstein) is more sensible.

It has been shown that in laparo-endoscopic hernia surgery, operative morbidity is dependent on surgeons' experience, and that significantly more complications are noted in surgeons' first 100 cases (10). In our case, the surgery was performed by a very experienced surgeon with more than 1,000 interventions. This fact demonstrates that major complications can occur even to very experienced surgeons. But in such cases their experience can help the surgeon to detect and handle the situation appropriately.

Severe hemorrhagic complications may be life-threatening. Fortunately, they are extremely rare in laparo-endoscopic inguinal hernia repair (11–14). In most cases, hemorrhage occurs as a result of an injury of the corona mortis, a communicating vessel between the obturator and external iliac vessel. The intraoperative detection of injuries of the corona mortis vein or even of the iliac vein in TEP is very difficult. Due to the pressure conditions in the preperitoneal space as a closed compartment, bleeding does not occur immediately, although the lumen of the vein can be seen (Figures 1, 2). In general, in TEP procedure the preperitoneal space is inflated by 12–14 mmHg CO_2_. On the one hand, this pressure creates enough space for adequate preparation, on the other hand it causes a pressure-associated compression on venous structures like the iliac vein or the venous corona mortis, leading to overlooked bleedings especially in the early postoperative period (15).

In TEP, the preperitoneal space is an independent compartment, that has a smaller surface area than the intraabdominal space. Therefore, according to the physical definition of pressure (*P* = F/A), in our opinion the pressure can be adapted more easily in this small compartment, and bleeding can be compressed faster than in the intraabdominal area, where hemorrhage can result in rapid blood loss.

During TEP hernioplasty, a decrease of the preperitoneal pressure to 8 mmHg can help to identify blood vessels or potential bleedings which might be missed at higher pressures (16). The adjustment of the preperitoneal pressure with the PEEP between 5 and 12 mmHg can be used in case of detected vessel injury and gives the surgeon enough time and good visibility to treat the injured vessel. Nevertheless, close communication between surgeon and anesthesiologist is necessary, especially in regard to the pressure adjustments (PEEP vs. preperitoneal pressure). The pressure ratios should be constantly adjusted to the operational situs, and should not be considered as absolute values. In this case, a vascular surgeon is not required. Moreover, in most cases the vascular surgeon would suture the vessel injury via laparotomy due to lack of expertise in laparo-endoscopic surgery, making a simultaneous hernioplasty very difficult.

Concerning the postoperative management of anticoagulation in such cases, a general recommendation cannot be given since there is no scientific data due to the rarity of this complication. The decision should be based on the individual clinical situation.

The possibility of adaption of the preperitoneal pressure with the PEEP is in our opinion one of the advantages of TEP over TAPP, where the larger space of the capnoperitoneum does not allow for the fast and adaptive pressure-related bleeding compression needed in this situation. Taking this into account, the question arises if the recommendation of the IEHS guidelines to perform TAPP rather than TEP in case of hernia recurrence after previously failed TAPP/TEP can be sustained (3). We would therefore prefer the recommendations of the Hernia Surge guidelines in which the choice of operation technique after a failed anterior and posterior repair should depend on patient- and surgeon-specific factors (2). In our opinion the TEP procedure is superior to TAPP at least with regards to the management of potentially life-threatening vessel injuries.

We would like to emphasize that this case presentation does not highlight the indication for TEP in patients with previous lower abdominal surgery. Certainly, in this specific patient an open surgery like Lichtenstein might have been the favorable option, and the complication might have been avoided. However, it has already be presented that TEP repair can successfully be performed after open abdominal surgery (17). The IEHS and the EAES guidelines also recommend that experienced surgeons can opt for a minimally invasive procedure in patients with previous pelvic operations (2, 3, 18).

In summary, this case highlights two clinical issues: TEP repair in patients with inguinal hernia and previous lower abdominal surgery is challenging but can be performed successfully. In case of major vein injury in TEP, the adjustment of the preperitoneal pressure can be used for endoscopic supply without performing a laparotomy.

## Data Availability Statement

The original contributions presented in the study are included in the article/supplementary material, further inquiries can be directed to the corresponding author/s.

## Ethics Statement

Ethical review and approval was not required for the study on human participants in accordance with the local legislation and institutional requirements. The patients/participants provided their written informed consent to participate in this study. Written informed consent was obtained from the individual(s) for the publication of any potentially identifiable images or data included in this article.

## Author Contributions

NT wrote the manuscript. VU conducted the follow-ups with the patient and obtained informed consent from the patient. TC revised, formatted, and submitted the manuscript. AM and DW conducted the surgery. All authors contributed to the article and approved the submitted version.

## Conflict of Interest

The authors declare that the research was conducted in the absence of any commercial or financial relationships that could be construed as a potential conflict of interest.

## Publisher's Note

All claims expressed in this article are solely those of the authors and do not necessarily represent those of their affiliated organizations, or those of the publisher, the editors and the reviewers. Any product that may be evaluated in this article, or claim that may be made by its manufacturer, is not guaranteed or endorsed by the publisher.

## Abbreviations

ASA: American society of anesthesiologists
CO_2_: Carbon dioxide
ECG: Electrocardiography
PEEP: positive end expiratory pressure
TAPP: transabdominal preperitoneal hernioplasty
TEP: totally endoscopic preperitoneal hernioplasty.
